# Follow the Cuts in American Healthcare From 2001 to 2026: A Three-Ledger Analysis of Scrutiny–Control Inversion, Payment Restraint, Revenue Capture, and Official Blame

**DOI:** 10.7759/cureus.113678

**Published:** 2026-07-30

**Authors:** Andrew M Klapper, Anthony N Dardano, Michael Risin, Taylor Florio, Monali Mahedia, Martha Denavea

**Affiliations:** 1 Plastic and Reconstructive Surgery, Delray Medical Center, Delray Beach, USA; 2 Nursing/Revenue Cycle and Clinical Documentation, Floridian Institute of Plastic Surgery, Delray Beach, USA

**Keywords:** claims adjudication, cost containment, healthcare economics, health policy, medicare, medicare advantage, no surprises act, physician payment, scrutiny-control inversion, vertical integration

## Abstract

Background

Physicians are frequently portrayed as principal drivers of American healthcare spending, although they often lack control over the prices, benchmarks, ownership structures, and corporate revenue flows attached to the care they provide. We examined whether federal cost policy directs scrutiny and containment toward economic actors in proportion to their control over price and revenue.

Methodology

We conducted a purposive structured descriptive analysis of major federal payment and cost-containment policies from January 2001 through July 2026. Evidence was organized into the following three ledgers: who receives the cuts, who controls and captures the money, and who receives the blame. A transparent, nonexhaustive inventory compared physician, insurer, pharmaceutical, and device-sector policies by breadth, automaticity, inflation sensitivity, durability, and reversibility. Rhetorical framing was assessed through matched Centers for Medicare & Medicaid Services (CMS) communications issued during the 2025-2026 policy period, and each principal finding was classified under an explicit four-level evidentiary standard.

Results

Medicare physician fee-schedule updates increased approximately 14% from 2000 through 2023 while the Medicare Economic Index increased approximately 52%; through 2024, the gap was approximately 14% versus 56%. The conversion factor declined from $38.26 in 2001 to $33.40-$33.57 in 2026. Physician payment remained subject to annual administered pricing, budget neutrality, temporary congressional relief, and recurring valuation adjustments. Among the major provisions examined, corporate-sector restrictions were more selective or reversible: the device excise tax and health-insurer fee were repealed; the Inflation Reduction Act created a recurring negotiation framework whose first 10 prices took effect in 2026, with CMS estimating $6 billion in net Medicare savings had those prices applied in 2023 and $1.5 billion in beneficiary savings in 2026; and Medicare Advantage payments increased 5.06% for 2026 despite MedPAC-estimated payments of $76-$84 billion above fee-for-service equivalence. International comparisons found U.S. prescription drug prices at 278% of prices in 33 peer countries overall and brand originator gross prices at 422%. Federal audit and transparency data documented payer-side control over realized claim payment. In an illustrative matched comparison of three CMS communications, physician policy was framed through waste and efficiency, insurer policy through access and stability, and manufacturer policy through innovation and certainty.

Conclusions

Across the major federal policies examined, physicians faced continuous, automatic, and inflation-insensitive payment restraint despite limited control over administered unit prices. Sectors with greater control over prices, claims adjudication, market access, and revenue capture generally faced narrower, later, or more reversible restrictions and more protective official framing. This convergent pattern supports a descriptive Scrutiny-Control Inversion. It does not establish coordinated intent or prove that political spending caused the observed policy outcomes.

## Introduction

The physician occupies an unusual position in American healthcare economics [1]. The physician identifies disease, recommends treatment, performs procedures, and generates the clinical record from which claims arise. These visible actions make the physician appear to generate the resulting expenditure, even when pricing and revenue distribution are controlled elsewhere. Visibility, however, is not economic control. We argue that economic regulation directed at the wrong economic actor may produce progressively diminishing returns regardless of its intensity.

A physician may determine that hospitalization, imaging, surgery, medication, rehabilitation, or a device is clinically necessary. The physician generally does not set the resulting prices: Medicare physician payment is administratively determined under federal statute and rulemaking [2-7]; insurer payment levels and benchmarks arise from plan bidding, contracting, and annual rate setting [8]; and drug and device prices are set by manufacturers in each national market [9-11]. Nor does the physician control whether a payer authorizes, downcodes, reprices, delays, or denies the resulting claim [12,13]. Clinical initiation and economic control are therefore distinct.

Federal policy nevertheless subjects physician payment to annual repricing, budget neutrality, utilization review, reporting obligations, audits, sequestration, surprise-billing restrictions, and periodic reductions [2-4]. Exceptional charges and arbitration awards are readily personalized as evidence of systemic physician overpayment, while corporate payment streams are more often discussed through access, innovation, stability, and sustainability. The contrast is particularly visible when globally marketed products command higher U.S. prices while locally delivered physician services remain comprehensively price-constrained [2-11].

Physicians influencing utilization [2,3] and instances of problematic billing conduct are documented in the Results. The question is whether scrutiny and containment are distributed according to actual price control, market power, and revenue capture. We define the Scrutiny-Control Inversion as follows:

The Scrutiny-Control Inversion occurs when economic scrutiny and cost containment are directed primarily toward the actor most visible at the point of care rather than toward the actors exercising the greatest control over price formation, payment methodology, market access, and revenue capture.

We test the descriptive component of the construct through the following three ledgers: who receives the cuts, who controls and captures the money, and who receives the blame. Each ledger alone permits an alternative explanation; the contribution lies in examining whether they diverge in the same direction across the selected evidence.

The article makes five contributions: it names and defines the construct; it introduces the three-ledger method for testing it; it adds two control dimensions, i.e., claims adjudication and international price dispersion, that standard payment analyses omit; it demonstrates official framing asymmetry through a matched, same-agency, same-period comparison rather than an unmatched anthology of quotations; and it classifies every principal proposition under an explicit evidentiary standard, separating what is proven from what is supported, plausible, or unsupported.

Our objective is not to prove a coordinated campaign or a quid pro quo between political spending and legislation. It is to determine whether payment architecture, revenue distribution, and official rhetoric display a recurring divergence consistent with asymmetric economic scrutiny, while marking the stronger claims that remain unproven.

## Materials and methods

Study design and scope

We conducted a purposive, structured, descriptive policy analysis covering January 2001 through July 2026. The design evaluates institutional architecture and observable policy outcomes rather than individual intent. It is not a systematic review or a complete census of every federal healthcare provision. The policy census, formal media corpus, and legislator-level political analysis required for population-level or causal inference are specified under Future Research. The analysis was organized into the following three evidence domains: Ledger I, payment and cost-containment burden; Ledger II, price control and revenue capture; and Ledger III, official attribution and framing.

Selection and coding procedure

The unit of analysis was a discrete federal payment or cost-containment provision. Candidate provisions were identified from the primary agency, legislative, commission, and peer-reviewed sources described below and were supplemented through citation tracing. A provision was eligible if it altered national payment levels, administered prices, taxes or fees, rebates, benchmark construction, reporting burden, or financial relief for at least one study sector. We prioritized provisions with national reach, direct fiscal effect, multiyear duration, or central relevance to the three-ledger framework.

Each retained provision was characterized using a structured matrix consisting of affected sector, economic direction, breadth, automaticity, inflation treatment, duration, administrative burden, implementation delay, and subsequent suspension, narrowing, repeal, replacement, or other modification. The Appendix presents the complete pre-census inventory used in this analysis and permits each classification to be compared with the cited authority. The inventory remains purposive and nonexhaustive, was not independently dual-coded, and has no inter-rater reliability statistic. It therefore supports reproducible descriptive comparison of the selected provisions but not a population estimate of all federal healthcare policy.

Evidence sources

Sources included MedPAC reports and public meeting materials [2,3]; Centers for Medicare & Medicaid Services (CMS) physician fee schedule rules [4] and the published conversion factor history [5]; official CMS communications [6,7]; CMS rate announcement materials [8]; international pricing literature [9,10]; agency drug negotiation communications [11]; federal audits and claims denial transparency analyses [12,13]; federal statutes and implementing law [14,15]; drug negotiation pricing and selection materials [16,17]; the National Health Expenditure Accounts and their peer-reviewed analyses [18,19]; foundational and comparative health economics literature [20]; MedPAC payment policy reports [21]; corporate disclosures and peer-reviewed payment transparency analyses [22,23]; court records and reporting on procedural rulings [24]; federal lobbying and campaign finance research [25]; dispute resolution outcome studies and federal data reports [26,27]; benchmark methodology documents [28]; and the governing payment statutes [29,30]. Principal documents were identified through agency and legislative repositories and supplemented by citation tracing. Advocacy sources were not used as the sole support for load-bearing quantitative findings.

International pricing studies were included when they compared matched products or product categories across multiple high-income countries and reported reproducible gross, modeled net, or hospital-acquisition price ratios. Device findings were treated as narrower than pharmaceutical findings when product configuration, bundled support, or confidential contracting limited comparability.

Claims management evidence was included when it derived from federal audits, CMS-mandated transparency data, or documented court proceedings. Claims management examples were selected to represent three distinct control points, namely, authorization before care, adjudication after care, and out-of-network repricing. Allegations in unresolved litigation were identified as allegations, and procedural rulings were not treated as findings of liability.

Sector definitions

We separated the healthcare economy into the following four principal sectors: (1) physicians and independent professional practices; (2) commercial insurers, Medicare Advantage organizations, and pharmacy benefit managers; (3) pharmaceutical manufacturers; and (4) medical device manufacturers. Hospitals, health systems, private-equity-backed staffing organizations, insurer-owned physician groups, and other corporate medical entities were not automatically classified as physicians. This distinction is methodologically necessary because the term “providers” routinely combines independent clinicians with large corporate organizations possessing materially different economic power; conflating them reproduces the very substitution error under study.

Analytic dimensions

Cost-containment policies were assessed by frequency, breadth, automaticity, inflation sensitivity, duration, reversibility, payment direction, administrative burden, implementation delay, and later suspension, narrowing, replacement, or repeal. Revenue control was assessed by authority over unit price, benchmarks, prior authorization, claims classification and adjudication, out-of-network repricing, payment timing, market access, benefit design, ownership, vertical integration, and retained revenue.

Framing comparison

The rhetoric analysis was a matched case comparison, not a corpus study. We compared CMS communications issued during the 2025-2026 policy period for the Physician Fee Schedule, Medicare Advantage rate setting, and Medicare drug negotiation. Matching the speaker and period reduces institutional and temporal confounding, but three communications cannot establish the prevalence of a federal or media-wide framing pattern.

Evidentiary standard

We distinguish the following four levels of inference: proven descriptively, directly documented by government, statutory, peer-reviewed, or primary institutional evidence; strongly supported, based on convergent but incomplete evidence; plausible but unproven, consistent with the record but vulnerable to alternative explanations; and unsupported, requiring evidence not presently available. Lobbying expenditures, campaign contributions, temporal association, and sector-favorable outcomes were not treated as proof of political causation. The standard is applied to each principal proposition at the end of the Results.

## Results

Ledger I: Who receives the cuts?

Medicare Physician Payment Has Been Continuously Compressed Relative to Practice Costs

MedPAC reported that cumulative Medicare physician fee-schedule updates increased approximately 14% from 2000 through 2023, while the Medicare Economic Index (MEI), the government’s official measure of physician practice-cost inflation, increased approximately 52% [2]. Subsequent MedPAC analyses extended the divergence through 2024, with cumulative updates remaining near 14% against cumulative MEI growth approaching 56% [3]. Reported MEI growth already incorporates a built-in adjustment for economy-wide productivity; the 52-56% figures therefore cannot be discounted on productivity grounds, because the discount is embedded in the index itself [2].

The divergence is prospective as well as retrospective. Statutory updates of 0.25% (most clinicians) and 0.75% (qualifying alternative payment model participants) remain below MedPAC-projected MEI growth of roughly 2.2% annually through 2034, producing an embedded, automatic real reduction of approximately 1.5-2 percentage points per year unless Congress affirmatively intervenes [2,3].

**Figure 1:**
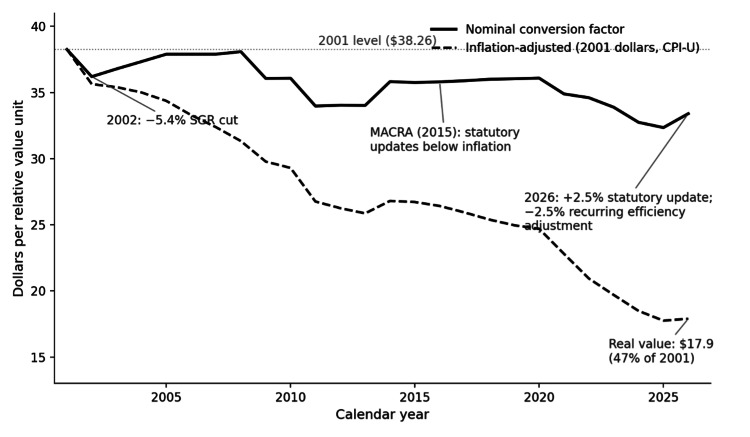
Medicare physician fee schedule conversion factor, 2001–2026: nominal and inflation-adjusted values. Medicare physician fee schedule conversion factor from 2001 through 2026 in nominal dollars and inflation-adjusted 2001 dollars. Inflation-adjusted values were calculated by multiplying each year’s nominal conversion factor by the ratio of the 2001 Consumer Price Index for All Urban Consumers (CPI-U) to the corresponding annual CPI-U. Annual CPI-U averages were used through 2025. The 2026 CPI-U value of 330.6 is an author projection based on 2.4% growth from the 2025 annual average; the resulting 2026 real-value estimate is therefore illustrative rather than an observed Bureau of Labor Statistics value. Conversion factors reflect values in effect on January 1; midyear revisions occurred in 2010, 2015, and 2024. The 2026 value of $33.40 applies to clinicians outside qualifying advanced alternative payment models; the qualifying-participant conversion factor is $33.57. Sources: Centers for Medicare & Medicaid Services physician fee schedule final rules; American Medical Association conversion-factor history; Bureau of Labor Statistics CPI-U series; author calculation for 2026.

The Medicare conversion factor was $38.26 in 2001. For 2026, the finalized conversion factors were $33.57 for qualifying advanced alternative payment model participants and $33.40 for all other clinicians [4,5]. The national conversion factor was therefore approximately 12.6% lower in nominal dollars in 2026 than in 2001, across a quarter century in which the general price level nearly doubled. Deflated by the Consumer Price Index, the 2026 conversion factor retains roughly 47% of its 2001 purchasing power (Figure 1); adjusted instead for practice-cost inflation, the American Medical Association estimates a decline of approximately 33% over the period [5].

Among the major payment streams examined, we identified no comparable national federal unit price that remained below its 2001 nominal level in 2026 (Appendix); confirming this negative exhaustively is a specified task of the complete policy census.

Temporary Legislative Relief Operates Within Permanent Administrative Restraint

The 2026 fee schedule is a live specimen of the payment architecture. Congress enacted a temporary 2.5% physician-payment increase for calendar year 2026 [4]. In the same cycle, CMS finalized a first-ever “efficiency adjustment” of -2.5% applied to the work relative-value units and intraservice times of most non-time-based services, procedures, imaging interpretation, and diagnostic tests, affecting more than 7,000 billing codes, to be revisited every three years against MEI-based productivity assumptions, alongside a practice-expense methodology change redistributing payment away from facility-based professional services [4,6,7].

The conversion factor increase and the efficiency adjustment operate through different mechanisms and did not affect every physician equally. The simultaneous efficiency adjustment nevertheless reduced the specialty-level benefit of the statutory increase for many procedure-based services, illustrating how temporary conversion factor relief can coexist with structural reductions elsewhere in fee schedule valuation. Positive legislative relief is temporary and must be affirmatively re-enacted, while efficiency adjustments, budget neutrality, and relative-value redistribution are built into recurring administrative operations. Physicians must repeatedly petition for protection from reductions generated automatically by the existing system; when temporary protection expires, the reduced baseline remains. The pattern is not new: of the annual cuts generated by the Sustainable Growth Rate formula between 2002 and 2014, only the first (-5.4% in 2002) was allowed to take full effect, with every subsequent cut deferred by temporary patches whose costs compounded until the formula’s repeal in 2015, whereupon Medicare Access and CHIP Reauthorization Act (MACRA) replaced it permanently with sub-inflationary statutory updates (Appendix).

Corporate-Sector Restrictions Have Frequently Been Narrower or Reversible

The Affordable Care Act (ACA) imposed a 2.3% excise tax on certain medical devices; the tax was suspended through repeated moratoria and permanently repealed effective 2020. The ACA’s annual health insurance provider fee was likewise suspended repeatedly and permanently repealed after 2020 [14]. These instruments are not economically identical to an administered professional fee schedule, taxes on corporate revenue and administered service prices operate differently, but the legislative record demonstrates that major federal financial restrictions directed at concentrated corporate sectors proved politically reversible, while, among the provisions examined, no major physician-payment restraint was removed without replacement by another durable administered mechanism during the study period.

Direct Medicare pharmaceutical price negotiation under the Inflation Reduction Act represents meaningful cost containment. The first negotiated prices took effect in 2026 for 10 drugs, with implementation phased and product-specific [15]. Medicare Advantage organizations operate under a separate annual rate-setting system; CMS finalized an average 5.06% increase in Medicare Advantage payments for 2026 [8].

The Inflation Reduction Act Was a Structural Breakthrough Despite Its Limited Initial Scope

The limited initial scope of Medicare drug negotiation should not obscure the Inflation Reduction Act’s institutional significance. The law gave Medicare direct negotiating authority for selected high-expenditure, single-source drugs for the first time. CMS estimated that, had the first 10 negotiated prices applied to 2023 utilization, net Medicare spending would have been approximately $6 billion lower, a 22% aggregate reduction for the selected drugs, and projected approximately $1.5 billion in beneficiary out-of-pocket savings in 2026 [16]. Negotiated prices remain in effect while products stay in the program and are updated annually.

The program is an expandable pricing architecture rather than a one-time intervention. CMS selected 15 additional drugs for prices effective in 2027; the statute authorizes up to 15 additional drugs in the third cycle and up to 20 in each cycle thereafter [17]. This is important counterevidence to any claim that pharmaceutical manufacturers are immune from federal cost containment. The contrast is not that pharmaceutical restraint is nonexistent. It is that physician services have been comprehensively and automatically repriced each year for decades, whereas comparable authority over pharmaceutical prices required new legislation, began with a limited product set, and is being implemented incrementally.

Ledger I Finding

Among the major federal provisions examined, physician payment demonstrated the most continuous, broad, automatic, and inflation-insensitive containment architecture. Corporate sectors faced meaningful regulation, but the principal restrictions examined were more often delayed, narrowed, product-limited, suspended, repealed, or offset by substantial payment growth.

Ledger II: Who controls and captures the money?

Physician Unit Prices Are Not the Principal Source of Healthcare Price Growth

National health expenditures reached approximately $4.87 trillion in 2023. Physician services proper accounted for approximately $721.7 billion (14.8%); the broader physician and clinical services category, approximately 20%; hospital care, approximately 31%; and retail prescription drugs, approximately 9%. The net cost of health insurance and government administration exceeded $300 billion (6.2%); financing, administration, and intermediation rather than direct clinical care [18,19].

CMS estimated physician service price growth at approximately 0.6% in 2023, against economy-wide inflation of approximately 4.1% [19]. Growth in the category therefore reflected utilization, intensity, employment structure, and other non-price factors rather than rising physician unit prices. Total spending on physician services can rise while the amount paid per standardized unit of physician work stagnates or declines in real terms. Physician service unit-price growth was not a major contributor to national healthcare price growth in 2023, despite continued public emphasis on physician overcharging, a finding consistent with the long-standing observation that American healthcare spending is distinguished by its prices rather than its utilization [20].

International Price Dispersion Reveals Product-Side Economic Control

International comparisons provide a direct test of whether product prices primarily reflect production cost or market-specific pricing power. A RAND analysis using 2022 data found that manufacturer gross prescription drug prices in the United States were 278% of prices in 33 comparison countries across all drugs. For brand-name originator drugs, U.S. gross prices were 422% of comparison country prices; after adjustment for manufacturer rebates, U.S. net brand prices remained more than three times as high. The United States accounted for 62% of sales, but only 24% of prescription drug volume across the countries studied [9].

The evidence for medical devices is narrower because hospital acquisition prices are often confidential and product configurations or bundled support may differ. In a cross-national study of cardiac implants purchased by hospitals in the United States, United Kingdom, France, Italy, and Germany, U.S. hospitals paid approximately two to six times the lowest-country price, depending on the stent or pacemaker category; the authors attributed variation in part to differences in purchaser bargaining power and willingness to pay [10].

These differences cannot be explained by a product becoming more clinically effective after crossing the U.S. border. They are consistent with substantial jurisdictional price discrimination and market-specific pricing power: globally marketed products can be priced according to purchaser bargaining power rather than a universal production cost or therapeutic value. Physician labor is local and nontransferable yet nationally price-constrained; patented drugs and devices are globally transferable yet can command substantially higher prices in the U.S. market. This contrast strengthens the Scrutiny-Control Inversion by identifying a major price-setting function that lies outside physician control.

MedPAC-Estimated Medicare Advantage Payment Differences Approach the Size of the Entire Physician Fee Schedule

MedPAC estimated that Medicare paid Medicare Advantage organizations approximately $84 billion (20%) more in 2025 than comparable beneficiaries would have cost in traditional Medicare, attributable to favorable selection (≈$44 billion) and coding intensity (≈$40 billion). For 2026, after full phase-in of the revised risk-adjustment model, the estimate remained approximately $76 billion (14%) above traditional Medicare equivalence [21].

**Figure 2:**
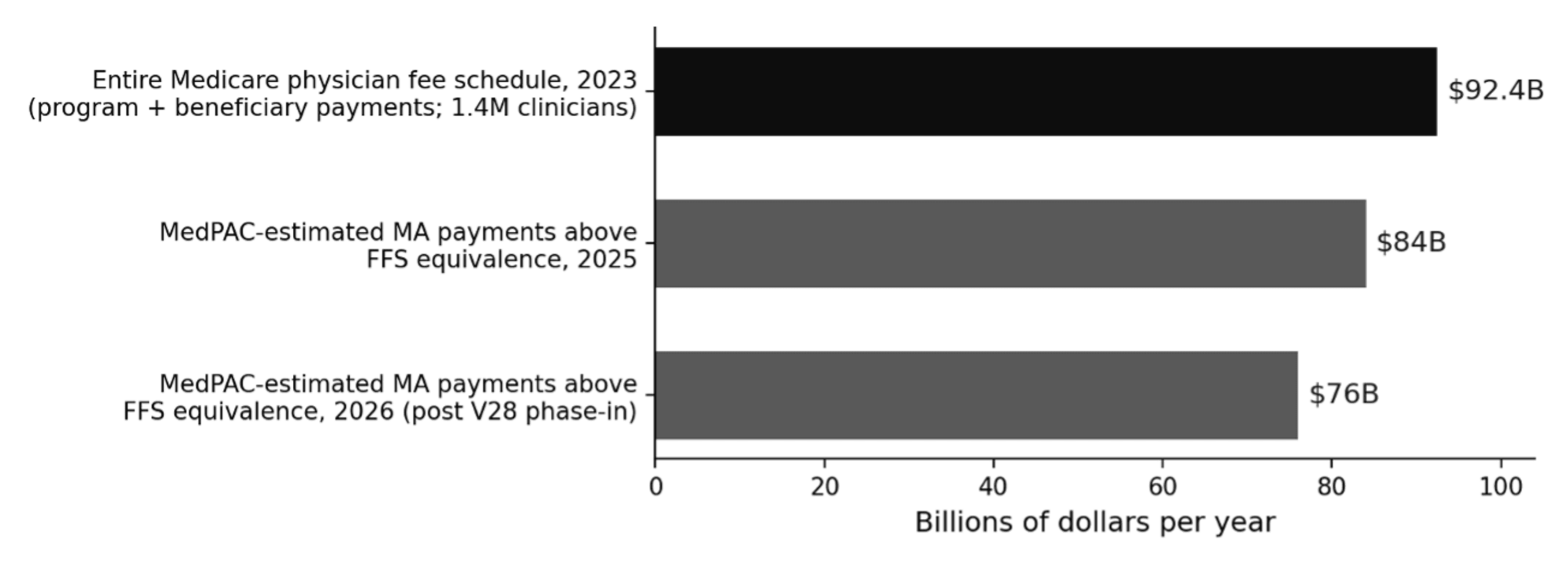
Scale comparison of medicare physician fee schedule payments and MedPAC-estimated Medicare Advantage payment differences. Scale comparison of annual payment flows and modeled payment differences, in billions of dollars. The bars represent different constructs, total fee-schedule payments (program plus beneficiary) and MedPAC-modeled payments above fee-for-service equivalence, and are juxtaposed to illustrate relative magnitude, not equivalence. The MedPAC estimates are contested and depend on assumptions about favorable selection, coding intensity, and counterfactual spending. Source: [21].

For scale: combined Medicare program and beneficiary payments under the entire physician fee schedule totaled approximately $92.4 billion in 2023, covering 1.4 million clinicians and 666 million patient encounters [21]. The MedPAC-estimated annual Medicare Advantage payment difference above fee-for-service equivalence therefore approaches the magnitude of total annual Medicare payment for all physician professional services in the United States (Figure 2). During the same policy period in which CMS titled its physician rule around cutting spending waste, it finalized the 5.06% average Medicare Advantage payment increase [7,8].

The methodology underlying these estimates is contested, and alternative analytic approaches produce different estimates; the figures should not be treated as undisputed accounting facts [21]. The magnitude comparison is illustrative rather than essential to the Scrutiny-Control Inversion. A materially lower estimate would weaken the numerical comparison with total physician fee schedule spending but would not eliminate the broader three-ledger finding, which also rests on independently documented physician fee compression, payer control over realized claim payment, international product price dispersion, the reversibility of major corporate-sector restrictions, and the matched CMS framing comparison. The structural thesis therefore does not depend on acceptance of the MedPAC estimate at its full reported magnitude.

Vertical Integration Separates Nominal Physician Spending From Physician Control

The category “physician spending” increasingly includes payments to practices owned by hospitals, private-equity firms, and insurers. The UnitedHealth Group reported that Optum Health employed or affiliated with more than 90,000 physicians in the period examined [22]. A 2025 Health Affairs analysis by Arnold and Fulton, using CMS payer price transparency data, found that UnitedHealthcare paid Optum-affiliated practices approximately 17% more than comparable non-Optum practices in the same markets, with larger differentials in markets where UnitedHealthcare held greater insurance market share. The authors interpreted the pattern as consistent with intra-conglomerate revenue transfer and a potential weakening of medical-loss-ratio constraints [23].

This finding exposes a measurement problem that the remainder of the analysis must respect: payment recorded as physician spending is not necessarily income controlled or retained by physicians. When an insurer pays an insurer-owned medical group, the transaction is classified as clinical expenditure while functioning, at least in part, as an internal corporate transfer. Treating such expenditure as evidence of independent physician enrichment misidentifies the economic beneficiary.

Claims Adjudication is an Economic Control Function

Insurer control extends beyond premium collection and network contracting to prior authorization, coding review, payment edits, out-of-network repricing, appeals, and payment timing. These processes determine not only whether care is covered but also whether a completed service is paid, how it is classified, and at what amount. They can prevent fraud, correct billing errors, and discourage low-value care; they can also transfer administrative cost, delay, and payment risk to clinicians.

Federal audit evidence demonstrates that errors within this control point are not merely hypothetical. In a stratified random sample of denials issued by 15 of the largest Medicare Advantage organizations, the Department of Health & Human Services Office of Inspector General (OIG) found that 13% of denied prior authorization requests met Medicare coverage rules and 18% of denied payment requests met both Medicare coverage and plan billing rules. OIG attributed cases to non-Medicare clinical criteria, unnecessary documentation demands, manual review errors, and system processing errors. The audit analyzed denials issued during a single week in 2019 and cannot be generalized to all plans or years, but it directly demonstrates that payer adjudication can delay medically necessary care and payment for services already delivered [12].

At a larger scale, a KFF analysis of CMS transparency data found that HealthCare.gov insurers denied 20% of in-network claims in 2023, with insurer-level rates ranging from 1% to 54%; fewer than 1% of denied claims were appealed. The data do not establish that most denials were improper, and they exclude much of the commercial market, but they demonstrate wide variation and the practical importance of the claims process [13].

Third-party repricing adds another layer. In 2025, a federal court allowed antitrust claims against MultiPlan and several major insurers to proceed, finding that providers had plausibly alleged a shared out-of-network repricing process that functioned more like mandates than recommendations. The defendants denied wrongdoing, and no final liability determination has been made [24]. The procedural ruling therefore does not prove price fixing; it shows that algorithmic repricing can be economically consequential enough to warrant antitrust scrutiny.

These mechanisms add a payer-side control layer to the Scrutiny-Control Inversion. The physician remains publicly associated with the expenditure because the physician delivered the care, while the insurer less visibly controls authorization, classification, payment level, appeal burden, and timing.

Large Corporate Healthcare Sectors Possess Substantial Organized Political Capacity

Peer-reviewed analysis found that pharmaceutical and health product interests spent approximately $4.7 billion on federal lobbying between 1999 and 2018, an average of $233 million per year, more than any other industry, plus $414 million in federal campaign contributions and outside spending and approximately $877 million in state political contributions, with contribution spikes timed to state drug pricing referenda [25]. Health professionals collectively accounted for approximately $1.7 billion of the $9.7 billion in total federal healthcare lobbying over the related period; that category includes professional associations and other health interests and should not be read as independent physicians alone [25]. The quantified evidence presented here is strongest for the pharmaceutical and health product sector; comparable sector-specific totals for insurers, hospitals, and device manufacturers are fragmented across lobbying and campaign finance databases and are targets of the legislator-level analysis specified under Future Research. These data demonstrate differences in organized political capacity. They do not establish that legislation was purchased or that contributions caused votes.

Ledger II Finding

Physicians exercised limited control over administered professional service prices, captured a minority share of national expenditures, and exhibited minimal measured unit-price growth in 2023, while insurers, manufacturers, hospitals, and vertically integrated conglomerates controlled substantial elements of pricing, contracting, claims adjudication, financing, and revenue distribution. The largest modeled payment difference examined was MedPAC’s estimate for Medicare Advantage, which was comparable in magnitude to the entire Medicare physician fee schedule. International comparisons further showed that globally marketed brand drugs, and, in a narrower cardiac implant sample, devices, were priced higher in the United States than in peer countries.

Ledger III: Who receives the blame?

Paired CMS Communications Used Different Rhetorical Frames Across Sectors

We conducted an illustrative matched comparison of a single-speaker CMS describing three payment streams during the 2025-2026 policy period (Table 1).

**Table 1:** Matched Centers for Medicare & Medicaid Services (CMS) framing across physician, insurer, and pharmaceutical payment Policy, 2025–2026. Paired official framing: the same agency and policy period, three sectors. Release titles are quoted verbatim as identifying titles of the cited public documents. Sources: CMS newsroom communications, 2025–2026 [6-8,11].

Payment stream	CMS communication, 2025–2026	Characteristic framing
Physicians (CY2026 PFS)	Proposed-rule release titled “CMS Proposes Physician Payment Rule to Significantly Cut Spending Waste”; final-rule release titled “CMS Modernizes Payment Accuracy and Significantly Cuts Spending Waste”	Waste; efficiency; overvaluation; payment accuracy; program integrity
Insurers (CY2026 MA rate announcement; +5.06% average payment)	Rate announcement emphasizes driving access to high-quality, affordable care; strengthening the program; and sustainability for current and future beneficiaries	Access; affordability; stability; choice; sustainability
Manufacturers (drug negotiation program)	Rule announcement pairs cost containment with manufacturer protection in its title, locking in lower prices while fostering innovation, and emphasizes certainty for manufacturers	Innovation; certainty; access; investment

Insurers and manufacturers are sometimes criticized, and physicians are sometimes praised. In this matched set, however, CMS framed comparable payment questions through materially different vocabularies: physician payment was linked to waste and efficiency; insurer payment, despite a large MedPAC-estimated payment difference, was linked to access and stability; and pharmaceutical restraint was linked, in the headline itself, to innovation protection. The paired design reduces institutional and temporal confounding, but the finding is limited to these communications and does not establish the prevalence of these vocabularies across CMS communications, the federal government, or national media.

The No Surprises Act Illustrates How Corporate Conduct Becomes Physician Blame

Federal independent dispute resolution (IDR) data show that initiating parties identified as providers or facilities won 81%, 85%, and 88% of payment determinations in 2023, 2024, and the first half of 2025, respectively, with median prevailing offers frequently exceeding the qualifying payment amount (QPA) by multiples of roughly 2.5 to 4.5 and awards totaling more than $2.2 billion above in-network benchmarks through 2024 [26,27]. These findings are legitimate matters of public concern.

Dispute volume and high-value awards, however, were highly concentrated: providers initiated over 99% of disputes, the top 10 initiating organizations accounted for roughly 71% of volume, and a small group of private equity-backed staffing and revenue cycle firms dominated both filings and outsized awards [27]. The economic conduct was therefore institutionally concentrated, not distributed across the physician workforce. This concentration creates a risk that broad labels such as provider abuse or physician overcharging will generalize institutionally concentrated conduct to the profession as a whole. It also creates a risk that an insurer-calculated benchmark will be treated as an independent market price.

The Qualifying Payment Amount Is Not an Independent Price

The QPA is generally calculated from a health plan’s median contracted rate for the same or similar service within a geographic area [28]. It is derived from insurer-held contracting data; it is not an independent government market survey, a physician cost measure, a physician charge, or a neutral adjudicated value. For the frequently disputed emergency service examined by Duffy and colleagues, the reported mean QPA was approximately 2.4 times Medicare, modestly below commercial in-network estimates of 2.5-2.7 times Medicare reported in prior literature; 6.3% of QPAs in that analysis fell below the Medicare-allowed amount [26]. These findings cannot be generalized to every code, specialty, payer, or geography. An award above the QPA may still be excessive, but the degree of excess cannot be inferred by silently treating an insurer-derived benchmark as an independent market-clearing price.

Outliers Require Denominators

An extreme physician charge, arbitration award, or utilization pattern establishes that an outlier exists; it does not establish prevalence or representativeness. Valid interpretation requires the number of physicians or entities involved, total eligible claims, median and interquartile payment distributions, case complexity, geographic variation, the insurer’s initial payment, professional payment as a proportion of total episode spending, concentration among repeat corporate entities, and the distinction between billed charges and actual payments. Absent those denominators, individual outliers generate public salience without establishing a profession-wide economic pattern.

Ledger III Finding

In the illustrative matched set of CMS communications, physician payment was framed through waste and efficiency, while insurer and manufacturer policy was framed through access, stability, innovation, and certainty. The recent IDR controversy was concentrated among a limited number of corporate entities and evaluated against an insurer-calculated benchmark. This establishes a paired official language asymmetry within the matched set; its prevalence across federal communications and national media remains unmeasured.

The three-ledger triangulation

Table 2 and Table 3 summarize the triangulation. Table 2 compares control over price formation and realized claim payment. Table 3 compares containment architecture, reversibility, observed payment patterns, and official vocabulary.

**Table 2:** Ledger II: Control over price formation and realized claim payment across healthcare sectors. Comparison of physician, insurer/Medicare Advantage, pharmaceutical manufacturer, and medical device manufacturer control over unit-price formation and realized claim payment. The table distinguishes clinical initiation from authority over contracting, network design, benchmark construction, prior authorization, claims editing, repricing, appeals, payment timing, formulary and benefit-design decisions, procurement, and related payment mechanisms. Sources: [2-17,21-28].

Dimension	Physicians	Insurers/Medicare Advantage	Pharmaceutical manufacturers	Device manufacturers
Control over unit price	Limited; Medicare prices administered since 1992	Contracting, networks, bids, benchmarks, and QPA data	Launch and market prices, subject to rebates and selected negotiation	Product pricing and contracting
Control over realized claim payment	Submit documentation; limited authority over authorization, coding edits, repricing, appeals, or payment timing	Prior authorization, claims edits, denials, appeals, out-of-network repricing, and payment timing	Indirect through formulary, rebate, and benefit-design decisions	Indirect through coverage, procurement, and contract terms

**Table 3:** Three-ledger comparison of containment architecture, reversibility, payment patterns, and official framing across healthcare sectors. Comparison of physicians, insurers/Medicare Advantage organizations, pharmaceutical manufacturers, and medical device manufacturers across the principal federal containment mechanism, breadth and automaticity, reversibility, observed price or modeled payment pattern, and characteristic official vocabulary. Read together with Table 2, the table summarizes the convergent relationship among unit-price control, realized claims-payment control, containment burden, and official framing. Sources: [2-17,19-23,25-30].

Dimension	Physicians	Insurers / Medicare Advantage	Pharmaceutical manufacturers	Device manufacturers
Principal federal containment	Annual PFS; budget neutrality; reporting; sequestration; recurring valuation changes	MLR rules; risk adjustment; insurer fee; transparency; benefit regulation	Rebates; inflation penalties; recurring selected negotiation	Excise tax; procurement pressure
Breadth and automaticity	Broad, annual, automatic; all covered services	Mixed; annual rate setting may raise or lower payment	Product-limited and phased	Tax-based; no national administered product price
Reversibility	Structural restraint retained; relief generally temporary	Insurer fee suspended and repealed; rates adjusted annually	Negotiation recent, phased, recurring, and litigated	Tax suspended and repealed
Observed price / modeled payment pattern	0.6% price growth in 2023; conversion factor below 2001 nominal level	MedPAC-estimated $84B (2025) and $76B (2026) above FFS equivalence; affiliated-provider payment differential	U.S. prices: 278% of peer-country prices overall; brand-originator gross prices 422%; adjusted net brand prices >3× peer prices	Cardiac implants: U.S. hospital prices approximately 2–6× the lowest-country prices in a five-country study
Default official vocabulary	Waste; efficiency; overvaluation; payment accuracy	Access; affordability; stability; choice; sustainability	Innovation; certainty; access; investment	Innovation; manufacturing; employment; access

Across the policies examined, containment intensity and durability were greatest for the sector with the least control over administered unit price, while sectors with greater control over pricing, claims adjudication, and revenue capture generally received more selective or reversible containment and more protective official framing.

Evidentiary classification of principal propositions

Applying the four-level standard specified in the Materials and Methods, Table 4 classifies each principal proposition of this study. The table is intended to prevent the descriptive findings from being read as stronger claims and the unproven claims from being attributed to the study.

**Table 4:** Evidentiary classification of the principal Scrutiny–Control Inversion propositions. Classification of the study’s principal propositions under the explicit four-level evidentiary standard defined in the Materials and Methods. “Proven descriptively” indicates direct documentation by government, statutory, peer-reviewed, or primary institutional evidence; “strongly supported” indicates convergent but incomplete evidence; “plausible but unproven” indicates consistency with the record but substantial vulnerability to alternative explanations; and “unsupported at present” indicates that the required causal or coordination evidence is not available.

Proposition	Evidentiary level	Principal basis and limits
Physician unit-price compression: 14% updates vs. 52–56% Medicare Economic Index (MEI); conversion factor below its 2001 nominal level	Proven descriptively	MedPAC series; Centers for Medicare & Medicaid Services (CMS) final rules; American Medical Association (AMA) CF history [2-5]
Corporate restriction reversibility: device tax and insurer fee suspended and repealed; no equivalent removal of physician administered-pricing architecture	Proven descriptively for the identified repeals; strongly supported for the cross-sector comparison	Public laws [14]; Table 5 (Appendix); the negative comparative claim awaits the complete census
*Inflation Reduction Act (*IRA) negotiation as recurring, expandable, but recent and product-selective containment	Proven descriptively	Statute; CMS savings estimates are agency counterfactual models [15-17]
Medicare Advantage payments above fee-for-service equivalence ($76–$84B/year)	Strongly supported	MedPAC modeled estimates; methodology contested [21]
International drug-price dispersion (278% overall; 422% brand gross; >3× net)	Proven descriptively (gross); strongly supported (net)	RAND matched comparison; net prices modeled for U.S. rebates [9]
Payer-side control over realized claim payment, with documented error rates	Proven descriptively (mechanism and audited samples)	OIG audit (1-week 2019 sample); KFF Marketplace transparency data; scope limits stated [12,13]
Intra-conglomerate payment differential to insurer-affiliated practices	Strongly supported	Single peer-reviewed transparency data analysis [23]
Matched CMS framing asymmetry across three 2025–2026 communications	Proven descriptively (for the matched set)	Verbatim titles and release text; prevalence beyond the set unmeasured [6-8,11]
Media-wide framing asymmetry reproducing official vocabulary	Plausible but unproven	Requires the formal media corpus
Fee compression accelerating loss of independent practice	Plausible but unproven	Mechanism economically coherent; causal evidence not established here
Political spending caused the observed policy asymmetries	Unsupported at present	Capacity documented [25]; causation requires legislator-level analysis
Coordinated intent (a designed “shell game”)	Unsupported at present	No coordination evidence; structural explanation suffices for observed pattern

## Discussion

The three ledgers identify a recurring misalignment between visibility and economic control. Physicians are personally attached to claims, procedures, prescriptions, and arbitration submissions. Corporate pricing, risk adjustment, rebates, internal transfers, facility fees, and administrative retention are distributed across institutions and accounting systems. The public therefore sees the doctor’s charge more readily than the economic architecture surrounding it.

Counterevidence

A credible analysis must state the evidence most likely to weaken its thesis more clearly than its critics would.

Total Physician Spending Increased Despite Unit-Price Compression

The same MedPAC analysis documenting the 14% versus 52% update gap shows that Medicare spending per beneficiary on fee schedule services increased approximately 101% over the period through volume and intensity [2]. Declining real unit prices did not prevent total spending growth. Physicians and organizations may respond through greater volume, service intensity, coding, ancillary revenue, consolidation, or employed practice. The construct does not deny spending growth. Its claim is narrower: administered physician unit prices are already tightly controlled, and compressing them further has not contained total spending.

Physician Compensation Remains High and Access Remains Stable

MedPAC reports median physician compensation of approximately $352,000 in 2023, nominal compensation growth from 2019 through 2023, stable beneficiary access, and near-universal Medicare participation [3,21]. This is not a physician poverty argument. High compensation does not establish control over unit prices, ownership, or corporate revenue. The unresolved question is whether income has increasingly depended on higher throughput, employment, commercial cross-subsidy, or loss of independent ownership.

Provider-Side IDR Extraction Is Real

Federal IDR data show provider-side entities achieving high win rates and awards well above qualifying payment amounts; these results cannot be dismissed. The concentration of disputes among large private equity-backed staffing and revenue-cycle organizations, however, makes disaggregation essential. Conduct by such entities, or by insurer-affiliated groups, should not be attributed to independent physicians or to the medical profession generally. Properly disaggregated, the episode demonstrates the substitution error rather than refuting the thesis.

Insurers and Manufacturers Face Meaningful Regulation

Insurers are subject to medical loss ratio rules, benefit mandates, risk-adjustment reform, network requirements, transparency obligations, antitrust scrutiny, and state regulation. Pharmaceutical manufacturers face Medicaid rebates, Medicare inflation penalties, selected negotiation, patent challenges, and formulary pressure. Device manufacturers face reimbursement uncertainty, procurement pressure, regulatory costs, and hospital bargaining. Corporate sectors are regulated. The difference lies in how often restraint is applied, how broadly it operates, whether it is automatic, and how easily it can be reversed. The medical loss ratio example is instructive: vertical integration may weaken the practical constraint imposed by medical loss ratio rules, and no comparable pathway exists on the physician side.

Claims Management Can Serve Legitimate Cost-Control Functions

Prior authorization, coding review, and claims editing can prevent duplicate billing, fraud, noncovered services, and low-value care. OIG noted that Medicare Advantage organizations approve the vast majority of requests, and neither Marketplace denial rates nor the MultiPlan procedural ruling establish that most denials or repricing decisions were improper [12,13,24]. The policy question is therefore not whether claims controls should exist, but whether criteria are transparent, error rates are monitored, appeals are accessible, algorithms are auditable, and compensation arrangements avoid rewarding unjustified underpayment.

Pharmaceutical Negotiation is Substantive, Not Symbolic

The Inflation Reduction Act created genuine and expandable pharmaceutical cost containment. It gave Medicare direct negotiating authority for selected high-expenditure, single-source drugs for the first time. CMS estimated that applying the first 10 negotiated prices to 2023 utilization would have reduced net Medicare spending by approximately $6 billion, or 22%, and projected approximately $1.5 billion in beneficiary out-of-pocket savings in 2026 [16]. CMS then selected 15 additional drugs for prices effective in 2027, and the statute authorizes additional annual cohorts [17]. These facts weaken any absolute claim that manufacturers escape direct price control. They do not erase the architectural contrast: pharmaceutical negotiation remains recent, product-selective, and phased, whereas physician payment restraint is comprehensive, longstanding, and automatic.

International Reference Pricing May Produce Strategic Responses

International reference pricing may produce strategic manufacturer responses, including altered launch timing, launch sequencing, foreign pricing, or product availability. These potential behavioral responses were not evaluated in this study and would require prospective policy modeling. Pharmaceutical and device innovation also involves research, regulatory, and product failure costs that are not captured by marginal manufacturing expense, a design consideration any reference pricing framework must accommodate. These considerations do not justify unlimited U.S. premiums, but they support using comparable high-income countries, verified net prices, access safeguards, and transparent adjustments rather than an unqualified worldwide lowest-price rule.

Clinical Initiation Is Not Economic Control

**Figure 3:**
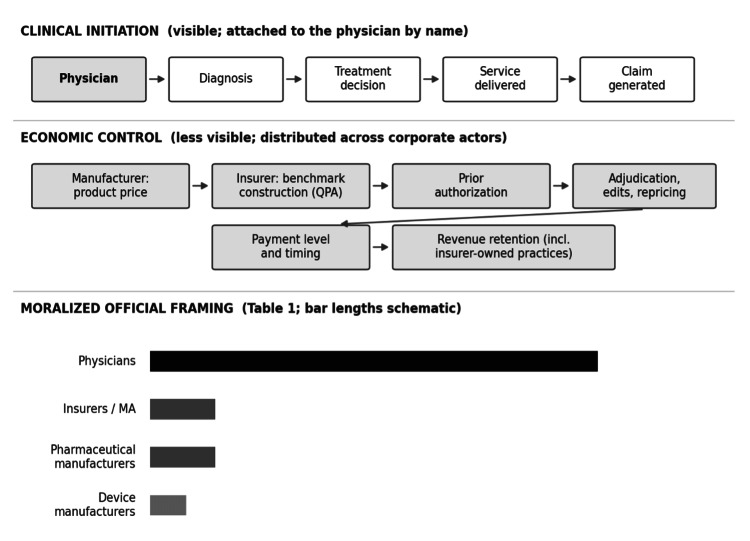
The Scrutiny–Control Inversion: Clinical visibility, economic control, and official framing. The upper panel shows the visible clinical pathway through which the physician initiates care and generates a claim. The middle panel shows the less visible chain of economic control over product price, benchmark construction, authorization, adjudication, payment timing, and revenue retention. The lower panel depicts the asymmetry in official framing documented in Table 1; bar lengths are schematic and do not represent measured frequencies.

Physicians influence utilization through diagnostic and therapeutic decisions [2]. Utilization and unit-price formation are distinct economic mechanisms; this study evaluates the latter without denying the importance of the former. A physician may determine that an intervention is clinically necessary. Economic control instead concerns who establishes the unit price, constructs the benchmark, controls network access and benefit design, owns the clinical platform, and retains the resulting revenue. Cost policy that collapses these functions risks targeting the visible clinical decision while leaving the larger price and ownership structure unchanged (Figure 3).

In the matched communications summarized in Table 1, the characteristic vocabulary applied to physicians was “waste,” “efficiency,” and “overvaluation”; the vocabulary applied to insurers and manufacturers was “access,” “stability,” “innovation,” and “certainty.”

Geographic Price Variation Is Evidence of Economic Control

The physician determines whether a drug or device is clinically indicated; the physician does not determine the national price imposed on the episode. When the same manufacturer accepts materially different prices for the same globally marketed product [9,10], the resulting expenditure reflects market structure and bargaining power in addition to clinical utilization. Attributing the full product expenditure to the physician who prescribed or implanted it therefore confuses clinical initiation with economic price formation.

Claims Adjudication Converts Financing Into Price Control

An insurer is often described as a financing intermediary, but claims adjudication gives it direct control over the realized price of care. The physician may determine that a service is necessary and complete it; the insurer can determine whether it was authorized, whether the code is accepted, whether the claim is repriced, whether documentation is sufficient, and when payment occurs [12,13]. This control is less visible than the physician’s clinical act but is economically decisive. Where insurer or intermediary compensation is linked directly or indirectly to reductions in allowed payment, claims management can become a revenue allocation mechanism rather than a neutral administrative function [24]. Whether these systems are used improperly across the market remains unresolved. Economically, however, adjudication is part of price formation.

Unit-Price Compression May Contribute to Corporate Consolidation

Persistent statutory payment updates below practice-cost growth [29] create incentives that may favor employment, acquisition, higher throughput, reduced uncompensated care, or narrower participation. These responses can preserve physician income while shifting ownership and bargaining power to hospital-, private equity-, or insurer-controlled organizations [22,23]. This study does not show that fee compression caused consolidation. The mechanism is economically plausible, however, and it fits the observed divergence between nominal physician spending and physician control. This mechanism may create a reinforcing feedback loop. Persistent professional fee compression can weaken independent practice viability and encourage employment or acquisition. Payments to the resulting hospital-, private equity-, or insurer-controlled practices may continue to be classified broadly as physician or provider spending even though ownership, bargaining power, and revenue retention have shifted to corporate entities. Growth in that aggregate spending category may then be cited to justify further professional fee restraint, accelerating the same consolidation that altered the category. This study does not establish that complete causal sequence; it identifies a testable pathway for longitudinal ownership and payment analysis.

The Shell Game Is Structural Before It Is Intentional

The evidence does not establish coordinated action by insurers, manufacturers, journalists, regulators, and legislators. Coordination is not necessary for a structurally useful narrative to persist: institutions repeat claims that advance their interests, reporters select visible examples, agencies use established vocabularies, and legislators respond to salient controversies and organized constituencies. The resulting pattern can function like a shell game even when no single actor designed it.

Political Capacity Is Consistent With the Inversion But Does Not Prove Causation

Pharmaceutical and health product interests possess substantial documented lobbying capacity [25], while comparable sector-specific estimates for insurers, hospitals, and device manufacturers require separate analysis; several corporate-sector restrictions also proved politically reversible. That association warrants formal testing, but it does not prove purchased outcomes. Contributions may follow ideology; lobbying may provide information and access; and repeal may reflect sincere concerns about employment, premiums, innovation, or product availability. Causal inference requires legislator-level analysis linked to specific bills, amendments, committee actions, and votes.

Policy implications

Require Economic Attribution Before Cost Containment

Before labeling a sector a cost driver, policymakers should establish who created the clinical need, who selected the service, who determined the unit price, who set the payment benchmark, who captured the revenue, and who could realistically alter total expenditure. A physician who recommends necessary care may influence utilization while exercising no control over the prices attached to the episode.

Separate Independent Physicians From Corporate Medical Entities

Federal reporting should distinguish independent practices, hospital-owned practices, private equity-backed groups, insurer-owned medical groups, management services organizations, and revenue-cycle or arbitration firms. The term “provider” is economically inadequate when it combines individual clinicians with multibillion-dollar corporate organizations.

Require Denominator Disclosure in Public Payment Reports

Reports describing physician outliers should disclose the number of physicians or entities involved, the eligible population, distributional payment data, actual payment rather than billed charges, professional payment as a share of episode spending, ownership structure, and concentration among repeat entities.

Identify the Origin and Limits of Payment Benchmarks

Government and media reports should state whether a benchmark is government-administered, insurer-calculated, contract-derived, charge-based, cost-based, survey-based, or adjudicated. The QPA created by the No Surprises Act [30] should not be described in a manner implying that it is an independent, market-clearing price.

Apply Parallel Payment Language Across Sectors

If above-benchmark physician payments are described as waste or overpayment, MedPAC-estimated insurer payments above traditional Medicare equivalence should be described in comparable economic terms and with the same caveats concerning methodology. If insurer payment increases are defended through access and stability, physician payment adequacy should be evaluated under the same criteria.

Require Claims Management Transparency and Appeal Accountability

Insurers should report denial, downcoding, and repricing rates by reason, service, plan, and provider ownership status; reversal rates; median decision and payment times; use of automated or third-party repricing systems; and compensation arrangements tied to claimed savings. Independent audits should test whether criteria conform to governing coverage rules and whether affiliated practices receive materially different treatment from independent practices.

Consider International Net-Price Parity for Globally Marketed Products

Policymakers could evaluate limiting what U.S. public payers pay for an identical patented drug, formulation, or device model relative to independently verified net transaction prices accepted by the same manufacturer in comparable high-income countries, with transparent adjustments for volume, taxes, distribution, and bundled services, and with access safeguards. Humanitarian or income-adjusted prices in lower-income countries should be excluded from any reference basket. The design questions, i.e., basket composition, rebate verification, and manufacturer behavioral responses, are specified as an empirical task under Future Research rather than resolved here.

Incorporate Inflation Into Physician Payment Policy

A nationally administered fee schedule that persistently trails practice cost inflation produces predictable, automatic real reductions. Permanent inflation-sensitive updates would not eliminate budget oversight; they would make explicit whether Congress intends a real cut, rather than allowing one to occur silently through inflation.

Future research

Six analyses are required to extend the descriptive finding into a complete empirical and causal program. The policy census should come first because its coded provisions define the units for subsequent media and political analyses.

Complete Federal Policy Census

Every discrete federal payment and cost-containment provision affecting the four sectors from 2001 through 2026 should be independently dual-coded for direction, breadth, duration, automaticity, inflation treatment, fiscal effect, implementation delay, administrative burden, and later modification or repeal. Inter-rater reliability and a normalized Cost-Containment Burden Index should be reported.

Formal Media Corpus Analysis

A prespecified corpus of national news, congressional material, agency communications, and trade association releases should test whether physician payment is disproportionately framed through abuse, gaming, overcharging, fraud, and windfall, and whether corporate payment is framed through access, stability, innovation, and certainty. Coding should also test benchmark provenance, denominator disclosure, ownership identification, and separation of independent physicians from corporate entities.

International Product-Price Audit

Matched drugs, formulations, packages, and device models should be compared using verified net transaction prices across a prespecified basket of high-income countries, with adjustment for rebates, volume, taxes, distribution, regulatory obligations, and bundled services. The analysis should quantify the U.S. premium and distinguish documented country-specific costs from jurisdictional price discrimination. Because device contracts are frequently confidential, federal procurement and disclosure authority may be required to obtain comparable data.

Claims Friction and Repricing Audit

Using CMS transparency files, plan data, provider remittances, audit findings, and court records, researchers should measure denial, downcoding, repricing, appeal, reversal, and payment delay rates by payer and service; identify use of third-party algorithms; compare affiliated and independent providers; and quantify associations with practice viability and consolidation. Allegations of intentional suppression should require contract, compensation, communication, or adjudicated evidence.

Legislator-Level Political Analysis

Campaign finance and lobbying data should be linked to sponsorship, amendments, committee action, and votes involving physician payment, the No Surprises Act, Medicare Advantage, drug negotiation, and repeal of the device and insurer fees. Models should control for party, ideology, committee position, industry exposure, election timing, and leadership. The design can establish association and, with suitable quasi-experimental variation, bounded causal inference.

Prospective Validation

The Scrutiny-Control Inversion framework should be applied prospectively to pending and future federal legislation to test whether it predicts which healthcare sectors receive cost-containment efforts, how durable those efforts prove, and how they are officially framed. Successful prediction would elevate the construct from descriptive pattern to tested theory; failed prediction would falsify it.

Limitations

This study is a purposive structured policy analysis, not a systematic census. The selected provisions were chosen for national reach, fiscal importance, duration, or relevance to the framework. Because inclusion depended partly on relevance to the proposed construct, selection bias cannot be excluded, and the findings should not be interpreted as a population estimate of all federal healthcare policy. The inventory was not independently dual-coded, and no inter-rater reliability statistic is available. The sectors and policy instruments are not economically identical. Medicare Advantage estimates depend on contested assumptions about selection, coding, and counterfactual fee-for-service spending. The matched CMS comparison demonstrates rhetorical differences in three communications, not speaker intent or media-wide prevalence. Lobbying data demonstrate political capacity, not purchased outcomes. Physician unit-price compression does not imply that total physician spending, volume, compensation, or access declined. Finally, the analysis does not deny physician overutilization, inappropriate billing, market power, or abusive conduct; it argues that such conduct must be measured with representative denominators and attributed to the responsible economic entity.

Finally, this analysis evaluates payment architecture rather than patient outcomes. It therefore cannot determine whether an alternative allocation of cost-containment effort would improve affordability, quality, innovation, or health outcomes; those are empirical questions for the research program specified above.

International drug comparisons rely principally on manufacturer gross prices with modeled adjustment for U.S. rebates; net prices are not systematically observable across countries. The device evidence is narrower and may be affected by differences in configuration, volume, taxes, distribution, and bundled support. The proposed international net price parity rule is a normative policy recommendation, not an outcome evaluated in this study. International reference pricing may also alter manufacturer launch sequencing, foreign pricing, or product availability; those behavioral responses were not evaluated here and would require prospective policy modeling. The OIG denial study used a one-week 2019 sample from 15 large Medicare Advantage organizations; KFF Marketplace data do not represent all commercial insurance and do not distinguish valid from invalid denials; and the MultiPlan claims remain unadjudicated allegations despite surviving a motion to dismiss.

## Conclusions

Across the major federal provisions examined from 2001 through 2026, the most continuous, automatic, and inflation-insensitive payment restraint was directed at physicians, who exercise limited control over administered professional prices. Major corporate sectors with greater control over prices, claims adjudication, market access, ownership, and revenue capture generally faced narrower, later, or more reversible restrictions. International comparisons further showed that globally marketed brand drugs, and a narrower sample of cardiac implants, carried higher U.S. prices than in peer countries. The Inflation Reduction Act’s recurring negotiation framework is meaningful counterevidence, but it remains recent and product-selective compared with decades of comprehensive annual physician repricing. In an illustrative matched set of three CMS communications, physician payment was framed through waste and efficiency, while insurer and manufacturer policy was framed through access, stability, innovation, and certainty. *This convergent inverse relationship among economic control, containment burden, and official scrutiny constitutes descriptive evidence of the Scrutiny-Control Inversion. *The finding does not prove a coordinated campaign against physicians. It establishes something narrower: across the provisions examined, federal cost containment tracked the most visible clinical actor more closely than it tracked measurable economic control over prices, claims adjudication, ownership, and revenue retention. The direct policy implication of these findings is that cost-containment legislation should begin with economic attribution: identifying, before payment is modified, which actor sets the price, constructs the benchmark, adjudicates the claim, owns the paid entity, and retains the revenue. Whether policy designed on that basis contains costs more effectively than the current approach is a testable question for the research program specified above.

## Appendices

**Table 5:** Pre-Census inventory of major federal healthcare payment and cost-containment provisions, 2001–2026. Structured inventory of the principal federal payment and cost-containment provisions examined in this study across physicians, insurers, pharmaceutical manufacturers, and medical device manufacturers from 2001 through 2026. Each provision is characterized by sector, policy direction, implementation mechanism, duration, and subsequent modification, suspension, repeal, or replacement. The inventory operationalizes the analytic dimensions defined in the Materials and Methods and supports the Results; it is not a complete census and does not replace the independently dual-coded federal policy analysis proposed under Future Research. Sources: [2-5,8-11,14,21,27-30] and the cited public laws. Policy dimensions represent structured author classifications and have not yet undergone independent dual coding.

Year	Provision	Sector	Direction	Automatic or legislated	Duration/Subsequent history
2002	-5.4% SGR formula update (Balanced Budget Act of 1997)	Physicians	Cut all PFS services	Automatic (formula)	Only SGR-generated cut ever fully applied
2003	Consolidated Appropriations Resolution override (+1.6%)	Physicians	Temporary relief	Legislated patch	One-year; formula debt accrues
2003	MMA §601: +1.5% updates for 2004–2005	Physicians	Temporary relief	Legislated patch	Two-year; no SGR rebasing
2005	Deficit Reduction Act: 0% update for 2006	Physicians	Freeze	Legislated patch	One-year
2006	TRHCA: 0% update 2007; creates PQRS reporting	Physicians	Freeze + reporting burden	Legislated patch	Reporting programs persist (later MIPS)
2008	MIPPA: +1.1% for 2009; e-prescribing incentives/penalties	Physicians	Temporary relief + burden	Legislated patch	One-year
2010	Five successive SGR patches within a single year	Physicians	Serial temporary relief	Legislated patches	Deferred cuts compound
2010	ACA §9008: annual branded prescription drug fee	Pharma	Sector fee	Legislated	In effect; fixed aggregate, apportioned
2010	ACA §9010: annual health insurance provider fee	Insurers	Sector fee	Legislated	Suspended 2017 and 2019; repealed effective 2021
2010	ACA (HCERA) §1405: 2.3% medical device excise tax	Devices	Sector tax	Legislated	Moratoria 2016–2017, 2018–2019; repealed effective 2020
2010	ACA medical loss ratio requirements (80/85%)	Insurers	Margin regulation	Legislated	Permanent; vertical integration may weaken the practical constraint
2011	Budget Control Act: 2% Medicare sequestration	Physicians and all providers	Across-the-board cut	Automatic	Suspended 2020–2022 (pandemic); resumed; repeatedly extended
2015	MACRA: SGR repeal; 0.5% updates 2016–2019; 0% 2020–2025; 0.75%/0.25% thereafter; MIPS	Physicians	Permanent sub-inflation updates + reporting	Automatic (statutory schedule)	Structural; no inflation indexation
2020	CARES Act/subsequent acts: sequester suspension; +3.75% PFS for 2021	Physicians	Temporary relief	Legislated patch	One-year; not built into baseline
2020	Further Consolidated Appropriations Act: device tax and insurer fee repeal	Devices; Insurers	Permanent relief	Legislated	Permanent
2020	CAA 2021, Div BB: No Surprises Act (balance-billing ban; insurer-computed QPA; IDR)	Physicians/facilities; Insurers	Payment constraint + arbitration	Legislated	Permanent; reshaped by litigation (TMA cases)
2021	Protecting Medicare and American Farmers from Sequester Cuts Act: +3% PFS for 2022	Physicians	Temporary relief	Legislated patch	One-year
2022	Inflation Reduction Act: drug negotiation (10 drugs effective 2026); inflation rebates; insulin cap; Part D redesign	Pharma	Selective containment	Legislated, phased	Recurring: 10 products (2026), 15 (2027), up to 15 in the third cycle, then 20 per cycle; litigated
2022	CAA 2023: +2.5% for 2023; +1.25% for 2024	Physicians	Partial temporary relief	Legislated patch	Cuts partially applied notwithstanding
2024	CAA 2024: +2.93% for March–December 2024 (partial offset of 3.37% cut)	Physicians	Partial temporary relief	Legislated patch	Partial-year; baseline cut retained January–March
2025	No patch enacted: 2.83% conversion-factor reduction takes effect	Physicians	Cut applied	Automatic	Full-year reduction
2024–26	MA risk-adjustment model V28 phase-in	Insurers	Payment-accuracy tightening	Administrative, phased	MedPAC-estimated payment differences persist ($76B, 2026)
2025	Reconciliation Act (H.R. 1): +2.5% PFS for 2026 only	Physicians	Temporary relief	Legislated patch	One-year
2025	CY2026 PFS final rule: efficiency adjustment of -2.5% (recurring, 3-year cycle); practice-expense site differential	Physicians	Recurring administrative cut	Administrative, recurring	Structural; reduces the specialty-level benefit of the legislated increase for many services
2025	CY2026 MA Rate Announcement: +5.06% average payment update	Insurers	Payment increase	Administrative, annual	Concurrent with MedPAC-estimated payment differences
2026	First IRA Medicare negotiated maximum fair prices effective (10 products)	Pharma	Selective containment	Legislated, phased	First prices implemented under the IRA negotiation program
